# The Release of Nitric Oxide Is Involved in the β-Arrestin1-Induced Antihypertensive Effect in the Rostral Ventrolateral Medulla

**DOI:** 10.3389/fphys.2021.694135

**Published:** 2021-06-18

**Authors:** Jia-Cen Sun, Xing Tan, Lian-Jie Ge, Ming-Juan Xu, Wei-Zhong Wang

**Affiliations:** ^1^Polar Medical Research Center and Department of Physiology, Naval Medical Center, Naval Medical University (Second Military Medical University), Shanghai, China; ^2^Department of Obstetrics and Gynecology, Changhai Hospital, Naval Medical University, Shanghai, China

**Keywords:** β-arrestin1, NO, hypertension, RVLM, ERK1/2

## Abstract

β-Arrestin1 is a multifunctional scaffold protein with the ability to interact with diverse signaling molecules independent of G protein-coupled receptors. We previously reported that overexpression of β-arrestin1 in the rostral ventrolateral medulla (RVLM) decreased blood pressure (BP) and renal sympathetic nerve activity (RSNA) in spontaneously hypertensive rats (SHRs). Nitric oxide (NO) is widely reported to be involved in central cardiovascular regulation. The goal of this study was to investigate whether NO signaling contributes to the β-arrestin1-mediated antihypertensive effect in the RVLM. It was found that bilateral injection of adeno-associated virus containing *Arrb1* gene (AAV-Arrb1) into the RVLM of SHRs significantly increased NO production and NO synthase (NOS) activity. Microinjection of the non-selective NOS inhibitor *N*-nitro-L-arginine methyl ester (L-NAME; 10 nmol) into the RVLM prevented the β-arrestin1-induced cardiovascular inhibitory effect. Furthermore, β-arrestin1 overexpression in the RVLM significantly upregulated the expression of phosphorylated neuronal NOS (nNOS) by 3.8-fold and extracellular regulated kinase 1/2 (ERK1/2) by 5.6-fold in SHRs. The β-arrestin1-induced decrease in BP and RSNA was significantly abolished by treatment with ERK1/2 small interfering RNA (ERK1/2 siRNA). Moreover, ERK1/2 siRNA attenuated the β-arrestin1-induced NO production, NOS activity, and nNOS phosphorylation in the RVLM. Taken together, these data demonstrate that the antihypertensive effect of β-arrestin1 in the RVLM is mediated by nNOS-derived NO release, which is associated with ERK1/2 activation.

## Introduction

It is well known that β-arrestin1 is an important modulator of G protein-coupled receptor (GPCR) desensitization and internalization (Nuber et al., 2016). Increasing evidence has proved β-arrestin1’s versatile protective roles in cardiovascular diseases including cardiac injury and cerebral ischemia by regulating certain cellular signaling pathways (Tang et al., 2015; Chen et al., 2017). Recently, β-arrestin signaling has been proved to be protective for lessening the tissue damage in the heart in the context of COVID-19 infection (Manglik et al., 2020), and β-arrestin1 has been considered as an essential protective effector in pulmonary arterial hypertension by activating Akt/mTOR signaling (Ning et al., 2020). In the central nervous system, β-arrestin1 signaling has been found to be involved in the processes of microglia dynamics regulation and maintaining hippocampal neurogenesis (Tao et al., 2015; Li et al., 2016). Our previous study has demonstrated the protective effect of β-arrestin1 on cardiovascular activity in the rostral ventrolateral medulla (RVLM) of hypertensive rats. It is well known that the RVLM containing the pre-sympathetic neurons plays a key role in maintaining sympathetic vasomotor tone and blood pressure (BP) (Huber and Schreihofer, 2016). However, the antihypertensive mechanism of β-arrestin1 in the RVLM needs to be further determined.

Nitric oxide (NO), synthesized by NO synthases (NOS) family, underlies the vasodilation of blood vessels and central functions as an important neurotransmitter in the brain (Chan and Chan, 2014). One typical brain region where NO may be of primary importance in central cardiovascular regulation is the RVLM (Mayorov, 2005), in which NO deficiency aggravates the impaired sympathetic baroreflex and increased sympathetic outflow in the state of hypertension or heart failure (Kishi et al., 2003; Wang et al., 2003). Previous studies have shown that augmentation of NO derived from neuronal NOS (nNOS) in the RVLM is able to blunt sympathetic overactivity in heart failure rats (Wang et al., 2003; Gao et al., 2008). In additional, the nNOS/NO signaling is also believed to mediate sympathoinhibitory effects induced by a range of stimulations including angiotensin-converting enzyme 2 overexpression, angiotensin II (Ang II) type 1 receptor blockers, and exercise training (Wang et al., 2007; Zheng et al., 2011; Sousa et al., 2015). Interestingly, it is suggested that there is a possible relationship between β-arrestin and NO signaling. For example, a novel role of β-arrestin1 has been demonstrated positively in the regulation of eNOS phosphorylation, making it useful for treating pulmonary hypertension and other vascular diseases (Carneiro et al., 2017; Ma et al., 2019). However, it still remains elusive what roles β-arrestin1 plays in nNOS regulation in the RVLM. Hence, the present study was aimed to investigate the responses of nNOS/NO to β-arrestin1 stimulation in the RVLM in hypertension.

Neuronal NOS phosphorylation is reported to be regulated by extracellular regulated kinase 1/2 (ERK1/2) activation in the carotid artery (Guan et al., 2015). Accumulated evidence has demonstrated that ERK1/2 is closely associated with β-arrestin1 regulation in HEK-293 and other cancer cells (Shenoy et al., 2006; Zheng et al., 2012; Suleymanova et al., 2017). Enhanced ERK1/2 phosphorylation-induced NO release in the RVLM leads to a hypotensive response by adenosine A (2A) receptor activation (Nassar and Abdel-Rahman, 2008), suggesting an important role of ERK1/2 signaling involved in BP regulation in the brain stem (Cheng et al., 2012, 2016). Therefore, we further confirmed the possibility that β-arrestin1-dependent ERK1/2 phosphorylation determines the NOS activation and NO production in the RVLM of hypertensive rats.

Thus, the present study mainly focused on β-arrestin1’s effects on NO release in the RVLM, and the following two points were examined: (1) if NO in the RVLM mediates the β-arrestin1-induced cardiovascular effects and (2) if ERK1/2 signaling pathway contributes to linking to β-arrestin1’s effects on NO production.

## Materials and Methods

### Animals

All the experimental SHRs and Wistar-Kyoto (WKY) rats were 16 weeks old and purchased from Sino-British SIPPR/BK Laboratory Animal Ltd. (Shanghai, China). The procedures were approved by the Institutional Animal Care and Use Committee of Naval Medical University and were carried out under the guidelines of the National Institutes of Health Guide for the Care and Use of Laboratory Animals. All steps were taken to minimize the animals’ pain and suffering.

### Construction of Vector-Arrb1 and Injection Into the Rostral Ventrolateral Medulla

As previously described (Sun et al., 2018), the serotype 9 adeno-associated virus (AAV-9) purchased from OBiO Technology Company (Shanghai, China) was used as the vector to transport the rat Arrb1 cDNA (Accession No. NM_012910). The vector that was labeled by green fluorescent protein and contained all sequence elements except the *Arrb1* gene was used as the control (AAV-GFP).

The experimental rats were anesthetized with continuous inhalation of isoflurane (3%) and placed in a stereotaxic frame (Shanghai Alcott Biotech, Shanghai, China), as previously described (Wang et al., 2014). Briefly, the dorsal surface of the occipital was symmetrically drilled with two holes after the skull was well exposed. According to the atlas of rats (Paxinos and Watson, 1998), AAV particles containing GFP or *Arrb1* gene fragment were slowly bilaterally injected into the RVLM (3.0 mm posterior to the lambda point, 2.0 mm lateral to the midline, and 9.5 mm deep to the skull surface) with a 32-gauge Hamilton syringe (5 μl) in 5 min, and the bilateral injection volume was 250 nl. After the incision was well cleaned and sutured, rats were administrated with 1,000 units of penicillin by muscle injection to prevent infection.

### Small Interfering RNA Experimental Protocol

Small interfering RNAs (siRNAs) were purchased from Gema Biotechnology Company (Buenos Aires, Argentina). Nine days after overexpression of β-arrestin1 in the RVLM, SHRs were anesthetized and received bilateral RVLM microinjections of either pooled ERK1/2 siRNA (0.3 μg in 0.7 μl 10 mM JetSITM) (Polyplus-transfection, New York, NY, United States). This dose was tested to be effective through reducing the total ERK1/2 and p-ERK1/2 expression in the RVLM of WKY, based on a previous study (Yu et al., 2016), and a scrambled siRNA was used as the control. SHRs that received different treatments above were then divided into four groups (AAV−GFP + CON siRNA; AAV−GFP + ERK1/2 siRNA; AAV−Arrb1 + CON siRNA; and AAV−Arrb1 + ERK1/2 siRNA).

### Measurements of Blood Pressure, Heart Rate, and Renal Sympathetic Nerve Activity

In conscious rats, BP and heart rate (HR) were continually measured by non-invasive tail-cuff system (ALC-NIBP, Shanghai Alcott Biotech) 7 days after AAV-Arrb1 was injected into the RVLM according to our previous study (Sun et al., 2018). In anesthetized rats (urethane 800 mg/kg i.p. and α-chloralose 40 mg/kg i.p.), levels of BP, HR, and renal sympathetic nerve activity (RSNA) were recorded by the PowerLab system (ADInstruments, Dunedin, New Zealand), as previously described (Tan et al., 2017a). The right femoral artery was catheterized to monitor BP and HR. The left kidney, renal artery, and sympathetic nerves were well exposed retroperitoneally. The renal sympathetic nerve was cut distally to avoid recording afferent activity and suspended on a pair of silver recording electrodes. As previously depicted (Peng et al., 2011; Becker et al., 2016), the background noise for sympathetic nerve activity was recorded 20 min before the rat was killed. The baseline RSNA in each rat was calculated as a percentage of maximal RSNA after detracting the background noise.

### Acute Microinjection of Nitric Oxide Synthase Inhibitor Into the Rostral Ventrolateral Medulla

According to our previous study (Tan et al., 2017a), the rats were anesthetized and placed in the stereotaxic instrument. After the occipital was well ground and cerebellum removed, the dorsal surface of the medulla was well exposed. Microinjection of the NOS inhibitor *N*-nitro-L-arginine methyl ester (L-NAME; 10 nmol/100 nl) into the RVLM (2.0–2.5 mm rostral and 2.0–2.2 mm lateral to the calamus scriptorius, 3.0–3.2 mm deep to dorsal surface of medulla) was performed with a multiple-barrel micropipette. The RVLM was functionally identified by a pressor response to L-glutamate (2 nmol) injection. Changes in BP, HR, and RSNA were continuously recorded by the PowerLab system.

### Total Nitric Oxide Production and Nitric Oxide Synthase Activity Detection

Measurement of the total NO production in the RVLM was based on our previous study (Tan et al., 2017b). Briefly, the RVLM tissues were punched and lysed in lysis buffer (tissue specific, purchased from Beyotime Biotechnology, no. S3090, Shanghai, China). After a certain centrifugation, the supernatants were retained for concentration analysis. Then, total NO production was measured as content of nitrate and nitrite using Nitrate/Nitrite Assay Kit (Beyotime Biotechnology, no. S0023) in reference to manufacturer’s instructions.

NO synthase activity detection was conducted according to a previous study (Wang et al., 2011). RVLM tissues were lysed and centrifuged to get the supernatants, which were then employed for NOS activity detection using the assay kit purchased from Nanjing Jiancheng Bioengineering Co., Ltd. (Nanjing, China; no. A014-1). NOS activity was measured and marked as U/mg protein, which means that one unit of NOS activity underlies the production of 1 nmol NO per minute per mg of tissue protein. In general, total NOS activity was calculated by the following steps: 20 μl supernatant was incubated with 200 μl of substrate buffer and then 10 μl reaction accelerator. Next, 100 μl of chromogenic agent was added in the fusions. After being completely mixed at 37°C for 15 min, 100 μl of clearing agent and 2 ml of terminal solution were added to stop the reaction; and final absorbance at 530 nm was obtained by automated micro plate reader. All the calculations were normalized according to the standard curve.

### Western Blotting Analysis

As previously described (Tan et al., 2017b), rats were killed, and the brains were quickly extracted and stored at −80°C. The RVLM tissues were punched in a freezing microtome (−20°C) and thoroughly lysed in cell lysate for 30 min at 4°C. After centrifugation for 20 min, the supernatant was left to measure the protein concentration by bicinchoninic acid (BCA) kit. Then, the protein samples (35 μg) were denaturalized and run on a 10% sodium dodecyl sulfate–polyacrylamide gel electrophoresis (SDS-PAGE) gel followed by transfer to polyvinylidene difluoride (PVDF) membrane (Millipore, Burlington, MA, United States). After being blocked with 5% non-fat milk in TBST buffer, the membranes were washed and incubated with anti-phospho-nNOS (no. 5583, S1416, abcam, Cambridge, United Kingdom), anti-nNOS [no. 4234, Cell Signaling Technology (CST), Danvers, MA, United States], anti-phospho-ERK1/2 (no. 4377, Thr202/Tyr 204, CST), anti-ERK1/2 (no. 4348, CST), anti−β-arrestin1 (no. 32099, abcam), anti-GAPDH (no. sc-47724, Santa Cruz Biotechnology, Dallas, TX, United States), and anti-α-tubulin (no. T6074, Sigma-Aldrich, St. Louis, MO, United States) primary antibodies overnight at 4°C. After being washed three times, the membranes were incubated with species-specific secondary antibodies conjugated horseradish peroxidase (IBA GmbH, Göttingen, Germany) and finally subjected to chemiluminescent agent (Millipore) to detect the target protein binding by GeneTools software (Gene Company, Shanghai, China). The expression of target proteins was normalized by control GAPDH or α-tubulin.

### Immunofluorescence

According to our previous description (Tan et al., 2017a), the fluorescence staining was detected by laser confocal microscopy (TCS-SP5, Leica Microsystems GmbH, Wetzlar, Germany). In brief, after being lethally anesthetized, the rats were perfused through the aorta with 0.9% saline and 4% paraformaldehyde in phosphate-buffered saline (PBS). Then, the brain was removed and post fixed in 4% paraformaldehyde for 24 h followed by replacement with 20% sucrose (constituted in PBS) for 12 h to dehydrate the brain. After swift freezing, the brain was dissected into 20-μm slices, which were then floated in PBS and blocked in 10% bovine serum albumin (BSA) (constituted in PBS) for 2 h. The brain slices were carefully washed three times and incubated with anti-phospho-ERK1/2 primary antibody (no. 4377, Thr202/Tyr 204, CST) overnight at 4°C. Fluorescein Affinipure goat anti-rabbit IgG (H + L) (no. 111095, Jackson ImmunoResearch Laboratories, Inc., West Grove, PA, United States) was performed as the secondary antibody to mark red fluorescence, while the HOECHST (Beyotime Biotechnology, no. C1018) was used to mark blue fluorescence. Finally, the slices were uploaded on slides and detected by the laser confocal microscopy (TCS-SP5, Leica, Wetzlar, Germany).

### Statistical Analysis

All data were examined as mean ± SE. Differences in β-arrestin1 expression between WKY and SHRs were analyzed by unpaired *t*-test. Differences in ERK1/2 siRNA silence efficacy and BP change in conscious rats were analyzed by one-way ANOVA with Dunnett’s multiple comparisons and repeated measurements with Turkey’s multiple comparisons tests. All the other differences in mean values were analyzed by two-way ANOVA with Turkey’s multiple comparisons tests. All the data were analyzed by GraphPad Prism (GraphPad Software, San Diego, CA, United States). *P* values of < 0.05 were considered statistically significant.

## Results

### β-Arrestin1 Overexpression Increased Nitric Oxide Production and Nitric Oxide Synthase Activity in the Rostral Ventrolateral Medulla of Spontaneously Hypertensive Rats

**FIGURE 1:**
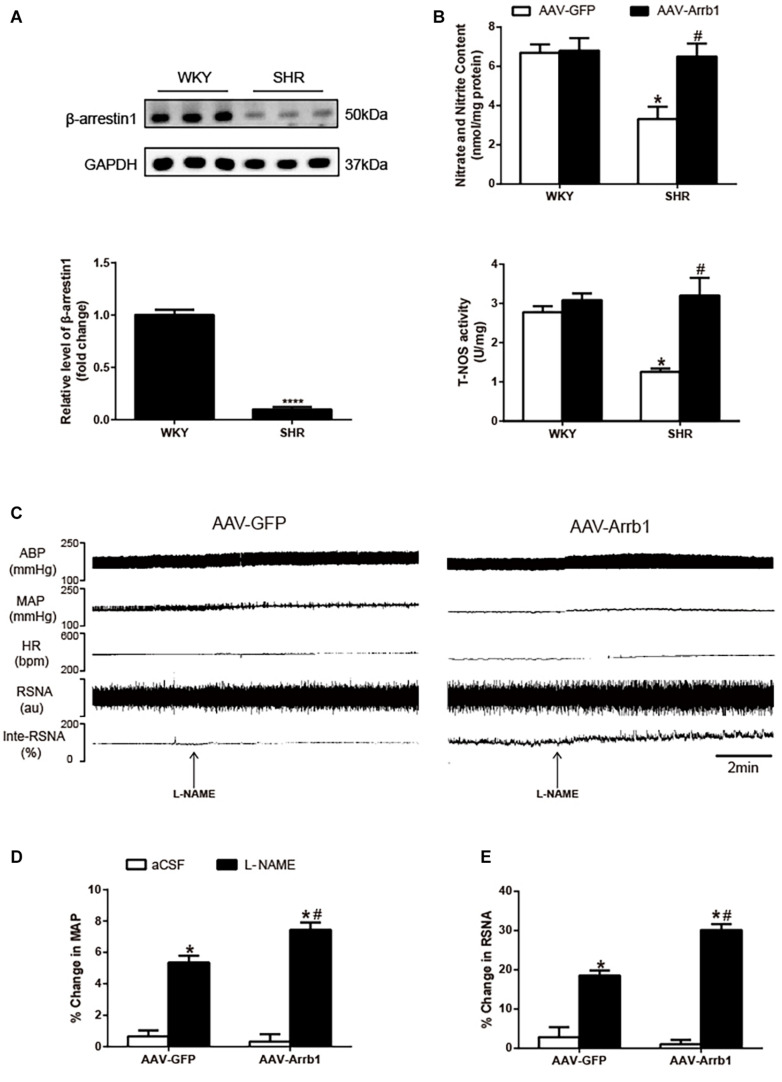
Effects of overexpression of β-arrestin1 on NO production and NOS activity in the RVLM. Representative gel bands (top) and quantification data (bottom) of β-arrestin1 **(A)**. **(B)** Relative changes of nitrate and nitrite content (top) and total NOS activity (bottom) in the RVLM in response to administration of AAV-GFP and AAV-Arrb1 in WKY rats or SHRs. *n* = 6 per group in A and *n* = 5 per group in B. ****P < 0.0001, *P < 0.05 vs. WKY; ^#^*P* < 0.05 vs. AAV-GFP. Original tracings **(C)** and quantification data **(D,E)** of BP, HR, and RSNA in response to unilateral microinjection of L-NAME (10 nmol) into the RVLM of SHRs pretreated with AAV transfection (AAV-GFP or AAV-Arrb1). *n* = 5 per group. *P < 0.05 vs. aCSF; ^#^*P* < 0.05 vs. AAV-GFP. MAP, mean arterial pressure; NO, nitric oxide; NOS, nitric oxide synthase; RVLM, rostral ventrolateral medulla; AAV, adeno-associated virus; GFP, green fluorescent protein; WKY, Wistar-Kyoto; SHRs, spontaneously hypertensive rats; BP, blood pressure; HR, heart rate; RSNA, renal sympathetic nerve activity; L-NAME, *N*-nitro-L-arginine methyl ester; aCSF, artificial cerebrospinal fluid.

As shown in Figure 1A, β-arrestin1’s expression in the RVLM was totally decreased in SHRs compared with WKY. The cardiovascular parameters of anesthetized rats were detected 2 weeks after β-arrestin1 overexpression in the RVLM (Table 1). Compared with the SHR + AAV-GFP group, SHRs that received β-arrestin1 overexpression showed a significant (*P* < 0.05) decrease in BP and baseline RSNA. As shown in Figure 1B, overexpression of β-arrestin1 significantly increased NO production and total NOS activity in the RVLM of SHRs compared with the control group. However, no significant changes of NO and NOS activity 2 weeks after β-arrestin1 overexpression were detected between WKY groups.

**TABLE 1:** Baseline levels of MAP, HR, and RSNA in anesthetized rats 2 weeks after transfection with AAV-GFP or AAV-Arrb1 in the RVLM.

	WKY		SHR	
	AAV-GFP	AAV-Arrb1	AAV-GFP	AAV-Arrb1
MAP (mmHg)	95 ± 4.34	104 ± 2.96	169 ± 4.97*	141 ± 5.84*^#^
HR (bpm)	358 ± 14.6	363 ± 18.6	421 ± 10.8*	404 ± 10.3
Basal RSNA (%Max)	12.1 ± 3.81	12.2 ± 1.94	33.9 ± 2.93*	12.3 ± 2.56^#^

Values are presented as mean ± SEM. n = 5 per group, *P < 0.05 vs. WKY; ^#^P < 0.05 vs. AAV-GFP. MAP, mean arterial pressure; HR, heart rate; bpm, beats/min; RSNA, renal sympathetic nerve activity; RVLM, rostral ventrolateral medulla; WKY, Wistar-Kyoto; SHR, spontaneously hypertensive rat; AAV, adeno-associated virus; GFP, green fluorescent protein.

We further assessed whether the β-arrestin1-induced decrease in BP and RSNA of SHRs was mediated by NO release in the RVLM. As shown in Figures 1C–E, microinjection of L-NAME (10 nmol) into the RVLM of SHRs pretreated with β-arrestin1 overexpression caused a significant increase in BP (%change: 5.35 ± 0.44 vs. 7.44 ± 0.48%, *P* < 0.05; pre-injection level of BP: 164 ± 6.65 vs. 129 ± 10.11 mmHg) and RSNA (%change: 18.53 ± 1.30 vs. 30.17 ± 1.53%, *P* < 0.05) compared with SHRs without β-arrestin1 overexpression.

### β-Arrestin1 Overexpression Enhanced Neuronal Nitric Oxide Synthase and Extracellular Regulated Kinase 1/2 Phosphorylation in the Rostral Ventrolateral Medulla of Spontaneously Hypertensive Rats

**FIGURE 2:**
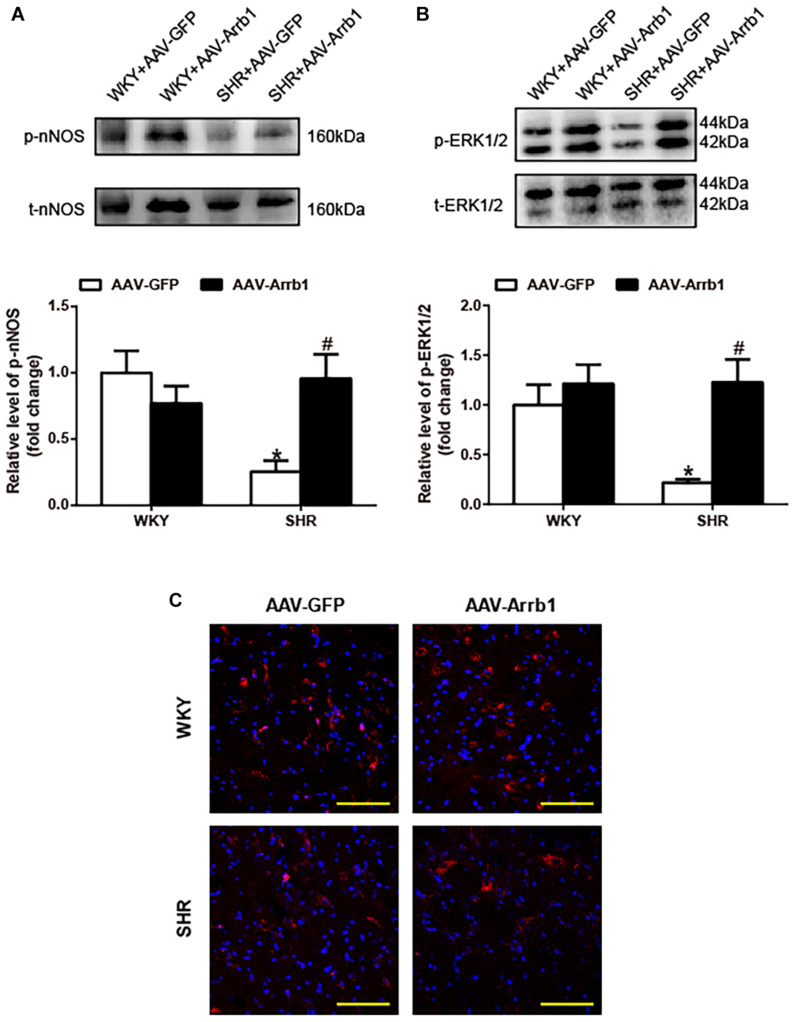
Effects of overexpression of β-arrestin1 on nNOS and ERK1/2 phosphorylation in the RVLM of SHRs. Representative gel bands (top) and quantification data (bottom) of phosphorylated nNOS **(A)** and ERK1/2 **(B)** in response to β-arrestin1 overexpression in the RVLM in SHRs. **(C)** Confocal microscopy photographs represent p-ERK1/2 (red fluorescence) and nucleus (blue fluorescence) in the RVLM. Scale bar = 200 μm. *n* = 5 per group in A and *n* = 4 per group in B, *P < 0.05 vs. WKY; ^#^*P* < 0.05 vs. AAV-GFP. Relative level, standardization of dividing phosphorylated values by total values. nNOS, neuronal nitric oxide synthase; ERK1/2, extracellular regulated kinase; RVLM, rostral ventrolateral medulla; SHRs, spontaneously hypertensive rats; WKY, Wistar-Kyoto; AAV, adeno-associated virus; GFP, green fluorescent protein.

As shown in Figure 2, overexpression of β-arrestin1 significantly increased the phosphorylation level of nNOS (≈3.8-fold, *P* < 0.05) in the RVLM of SHRs compared with the SHR + AAV-GFP group. Similarly, the phosphorylated level of ERK1/2 in the RVLM of SHRs was significantly decreased compared with that of WKY (*P* < 0.05). Similar to the change of nNOS, the phosphorylation level of ERK1/2 (Figure 2B) was notably increased (≈5.6-fold, *P* < 0.05) by β-arrestin1 overexpression in SHRs. Immunofluorescence staining also demonstrated that treatment with β-arrestin1 overexpression increased ERK1/2 phosphorylation in the RVLM of SHRs (Figure 2C). These data suggested that ERK1/2 in the RVLM was activated by β-arrestin1.

### The β-Arrestin1-Induced Reduction in Blood Pressure and Renal Sympathetic Nerve Activity in Spontaneously Hypertensive Rats Was Blunted by Extracellular Regulated Kinase 1/2 Small Interfering RNA in the Rostral Ventrolateral Medulla

**FIGURE 3:**
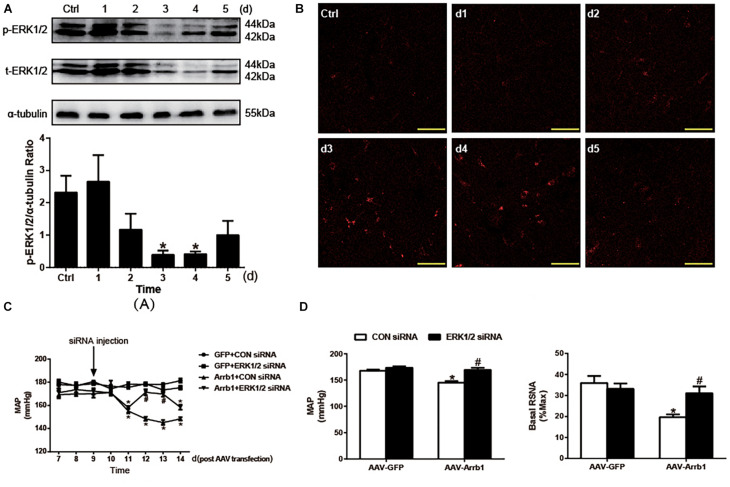
Effects of knockdown of ERK1/2 in the RVLM on the β-arrestin1-induced changes in BP and RSNA. **(A)** Representative gel bands (top) and quantification data (bottom) show the changes in phosphorylated and total ERK1/2 after treatment of ERK1/2 siRNA in the RVLM. *n* = 4 per group. *P < 0.05 vs. Ctrl. **(B)** Confocal microscopy photographs consecutively represent the potency and stability of siRNA-Cy3 (red fluorescence) in the RVLM from day 1 to day 5. **(C)** Levels of BP in the conscious groups (GFP + CON siRNA, GFP + ERK1/2 siRNA, Arrb1 + CON siRNA, and Arrb1 + ERK1/2 siRNA). The treatment of siRNA injection into the RVLM of SHRs was performed at day 9 post AAV transfection. *n* = 5 per group. *P < 0.05 vs. day 7 in the corresponding group; ^#^*P* < 0.05 vs. CON siRNA. **(D)** Changes in BP (left) and basal RSNA (right) of anaesthetized SHRs in response to siRNA injections. *n* = 5 per group. *P < 0.05 vs. AAV-GFP; ^#^*P* < 0.05 vs. CON siRNA. MAP, mean arterial pressure; Ctrl, rats that received transfection reagent without siRNA; ERK1/2, extracellular regulated kinase; RVLM, rostral ventrolateral medulla; BP, blood pressure; RSNA, renal sympathetic nerve activity; siRNA, small interfering RNA; GFP, green fluorescent protein; SHR, spontaneously hypertensive rat; AAV, adeno-associated virus.

In order to validate the critical role of ERK1/2 in the regulation of cardiovascular activity induced by β-arrestin1, ERK1/2 was knocked down by injection of specific ERK1/2 siRNA into the RVLM. As shown in Figure 3A, the silencing efficacy of ERK1/2 siRNA in the RVLM of WKY was examined by Western blotting. The expression of ERK1/2 phosphorylation in the RVLM was conspicuously decreased at day 3 after ERK1/2 siRNA injection compared with control treatment. Furthermore, we conducted the injection of a negative control siRNA (CY3)-labeled red fluorescence into the RVLM. As represented in Figure 3B, the intensity of red fluorescence at 3-day post-injection in the RVLM was mostly apparent compared with that without CY3 injection, which supports the result of ERK1/2 silencing efficacy above.

After identification of the efficacy of ERK1/2 siRNA in the RVLM, the β-arrestin1-ERK1/2 signaling pathway was verified by injection of siRNA into the RVLM of SHRs 9 days after AAV transfection. As a result, a significant increase in BP (148.4 ± 1.6 vs. 171.4 ± 2.5 mmHg, *P* < 0.05) was observed by ERK1/2 siRNA at day 12 after β-arrestin1 overexpression compared with that treated with the CON siRNA (Figures 3C,D). A similar change was found in BP (145.2 ± 2.6 vs. 169.2 ± 4.1 mmHg, *P* < 0.05) and baseline RSNA (19.7 ± 1.3 vs. 31.1 ± 3.2%max, *P* < 0.05) under the anesthetized state. These data indicated a critical role of ERK1/2 in the RVLM in the effect of β-arrestin1 on sympathetic hyperactivity and high BP.

**FIGURE 4:**
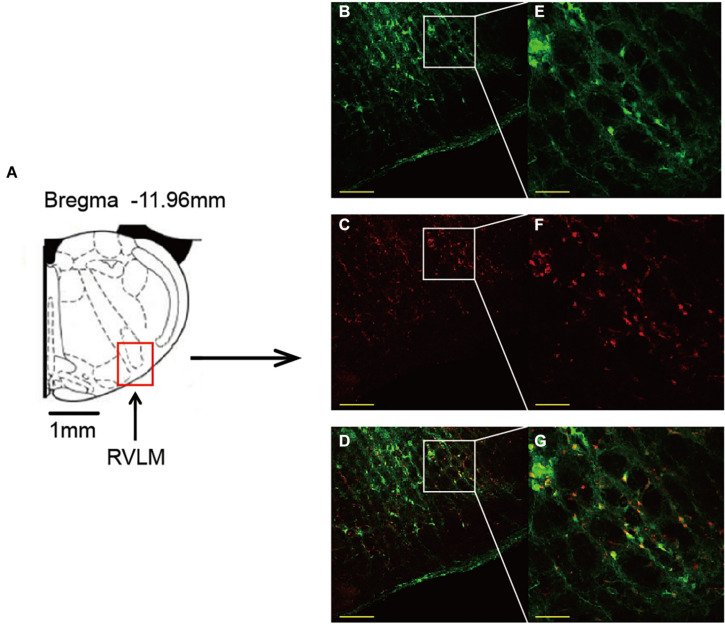
Confirmation of the same injection site of both AAV and siRNA delivered in the RVLM. **(A)** Standard rat atlas of the RVLM region. **(B,C)** Green fluorescent protein (GFP) and Cy3 (red fluorescence) confirmed in the RVLM. **(D)** Merged image of **(B,C)**. **(E–G)** Enlarged images of **(B–D)**. Scale bars = 200 μm in **(B–D)** and 50 μm in **(E–G)**. AAV, adeno-associated virus; siRNA, small interfering RNA; RVLM, rostral ventrolateral medulla.

As shown in Figure 4, same injection site of both AAV and siRNA delivered in the RVLM was confirmed using immunofluorescence staining.

### The β-Arrestin1*-*Induced Increases in Neuronal Nitric Oxide Synthase-Derived Nitric Oxide Production Were Blunted by Extracellular Regulated Kinase 1/2 Small Interfering RNA

**FIGURE 5:**
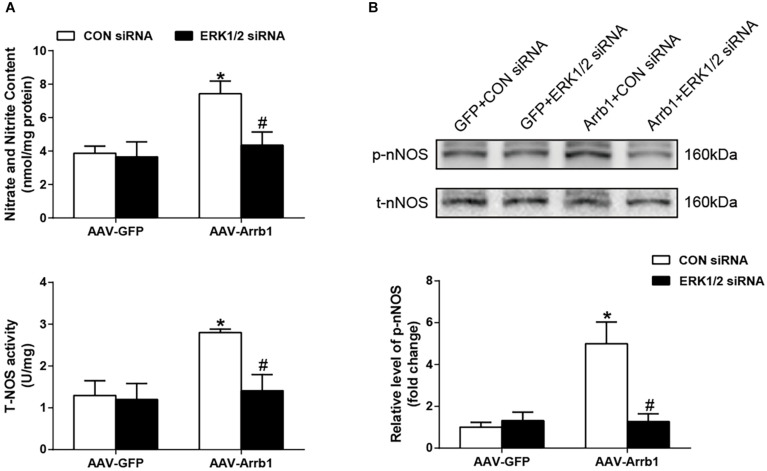
Effects of knockdown of ERK1/2 in the RVLM on the β-arrestin1-induced NO production and nNOS phosphorylation. **(A)** Relative changes of nitrate and nitrite content (top) as well as total NOS activity (bottom) in the RVLM among the four groups. **(B)** Representative gel bands (top) and quantification data (bottom) of changes in phosphorylated nNOS after treatment of ERK1/2 siRNA in the RVLM among the four groups. *n* = 5 per group, *P < 0.05 vs. AAV-GFP; ^#^*P* < 0.05 vs. CON siRNA. Relative level, standardization of dividing phosphorylated values by total values. ERK1/2, extracellular regulated kinase; RVLM, rostral ventrolateral medulla; NO, nitric oxide; nNOS, neuronal nitric oxide synthase; NOS, nitric oxide synthase; siRNA, small interfering RNA.

As shown in Figure 5, compared with the Arrb1 + CON siRNA group, administration of ERK1/2 siRNA significantly (*P* < 0.05) decreased NO level and NOS activity. The phosphorylation of nNOS expression was also decreased by ERK1/2 siRNA pretreatment. These data suggested that ERK1/2 was involved in interaction between β-arrestin1 and nNOS/NO signaling in the RVLM of SHRs.

## Discussion

This study mainly focused on the relationship between NO signaling and β-arrestin1 in the RVLM. The major findings were that (1) overexpression of β-arrestin1 in the RVLM significantly enhanced NO production and nNOS phosphorylation in SHRs; (2) β-arrestin1 overexpression in the RVLM increased ERK1/2 phosphorylation in SHRs; and (3) administration of ERK1/2 siRNA into the RVLM blocked the β-arrestin1-induced decrease in BP and increase in NO release as well as nNOS phosphorylation in SHRs. The current results suggest that NO signaling plays an important role in mediating the inhibitory effect on sympathetic tone and BP evoked by β-arrestin1 in the RVLM, which is associated with activation of ERK1/2 signaling pathway.

We have previously demonstrated a significant reduction in BP of SHRs 14 days after administration of AAV-Arrb1 into the RVLM (Sun et al., 2018). In this previous study, we have confirmed the accuracy and efficacy of β-arrestin1 overexpression in the RVLM by immunofluorescence and Western blotting. In the present study, we totally used the same animal models and experimental protocols. Although we did not further test the efficacy of β-arrestin1 overexpression in the RVLM, we also confirmed an inhibitory effect of β-arrestin1 overexpression in the RVLM on BP and RSNA in the anesthetized SHRs. Interestingly, a recent research has proved that RVLM overexpression of β-arrestin2, a homologous subtype with β-arrestin1, also ameliorated hypertension by enhancing the cannabinoid type 1 (CB1) receptor desensitization (Wang et al., 2017). Therefore, it is suggested that both β-arrestin1 and β-arrestin2 are involved in the central regulation of BP in the RVLM. In addition, we have detected a similar decrease of β-arrestin1 expression in the paraventricular nucleus (PVN) and nucleus of the solitary tract (NTS), rather than the cortex. As PVN and NTS are two important central sites involved in cardiovascular regulation, it motivates us to further determine the effects of β-arrestin1 on cardiovascular activities in these two regions.

Nitric oxide is well characterized for its vasodilatory role in cardiovascular regulation; and a number of studies have linked NO to cardiovascular activity in the RVLM (Machado et al., 2016; Lo et al., 2018). However, the complicated responses and potential mechanisms are not well established yet. It is important to note that NO in the RVLM may dose-dependently be able to elicit sympathoexcitation and sympathoinhibition (Chan et al., 2003; Huang et al., 2003, 2004). NO has been shown to reduce the release and formation of Ang II in the RVLM (Tagawa et al., 1999), and damage of this inhibitory effect of NO on Ang II may cause the neurogenic pathophysiological of hypertension (Zanzinger, 2002). Thus, considering that high concentration of Ang II in the RVLM contributes to sympathoexcitation and hypertension (Zhou et al., 2019), it is possible that maintaining of NO production in the RVLM is beneficial for cardiovascular dysfunction. β-Arrestins have been demonstrated to be involved in the regulation of sympathetic activity and BP in the RVLM (Wang et al., 2017; Sun et al., 2018). Moreover, it has been demonstrated that β-arrestin1 is critical for NO production in human vascular endothelia cells (Carneiro et al., 2017). However, the crosstalk between β-arrestin1 and NO in the RVLM has not been known yet. In the present study, we proved an important role of β-arrestin1 in evoking NO release in the RVLM of SHRs, which extended our knowledge of β-arrestin1’s effect on BP regulation under pathological conditions.

Nitric oxide production is regulated by NOS catalysis; and the three isoforms, nNOS, eNOS, and iNOS, are located in the RVLM (Chang et al., 2003). Although there are a variety of roles of NO from different NOS isoforms in the RVLM in cardiovascular regulation, nNOS is judged to be more prevalent in the regulation of basal sympathetic outflow and vasomotor tone (Chan et al., 2001). Moreover, neuroprotective effects of nNOS activation in the RVLM in response to upstream stimulation like resveratrol and heat shock protein 60 have been reported (Chan et al., 2007a; Cheng et al., 2016). Nevertheless, there is no consensus on the specific role of nNOS in facilitating or blocking cardiovascular dysfunction under pathological conditions. Importantly, in the current study, we found that overexpression of β-arrestin1 in the RVLM significantly increased nNOS phosphorylation. As upregulation of iNOS in the RVLM had long been accepted to cause sympathetic activation and hypertension (Kimura et al., 2005), it may be a limitation that the effects of β-arrestin1 on iNOS were not detected in this work. Based on our current data, it is implied that nNOS serves as the prioritized source of NO in response to the β-arrestin1-mediated sympathoinhibitory effect in the RVLM of hypertension.

NO synthase activity is affected by a diversity of cellular biochemical molecules such as PI3K/AKT, AMPK, RSK, and ERK1/2 (Chow et al., 2012; Cheng et al., 2013, 2014; Carneiro et al., 2017). It is reported that ERK1/2, a widely appreciated signaling molecule, mediates β-arrestin1-dependent signaling transduction (Shenoy et al., 2006). Moreover, ERK1/2 is reported to be critical for the regulation of BP in the brain stem (Ibrahim and Abdel-Rahman, 2012). The present study demonstrated that overexpression of β-arrestin1 significantly promoted ERK1/2 phosphorylation. As ERK1/2 activation in the RVLM could lead to sympathoinhibition or sympathoexcitation (Chan et al., 2005; Cheng et al., 2016), it was essential to define the role of ERK1/2 elevation by β-arrestin1 in control of the final BP response. It is noteworthy that ERK1/2 underlies contradictory responses elicited by upstream stimulators such as Ang II or α_2A_ adrenergic agonist (Chan et al., 2007b; Nassar and Abdel-Rahman, 2008). In the present study, we inhibited ERK1/2 using a specific ERK1/2 siRNA injection into the RVLM of SHRs. As a result, ERK1/2 inhibition abrogated the suppressor effect caused by β-arrestin1 in the RVLM, indicating that β-arrestin1 is tightly linked to ERK1/2 activation in the light of cardiovascular response. Moreover, no changes in BP and RSNA were observed in the deficiency of ERK1/2 in the RVLM free of β-arrestin1 overexpression. Collectively, our findings suggest that β-arrestin1-induced ERK1/2 activation certainly contributes to cardiovascular response. A previous study has demonstrated that overexpression of β-arrestin1 facilitates and solidifies ERK1/2 activation in the cytoplasmic pool but inhibits ERK1/2-dependent transcription in the nucleus (Tohgo et al., 2002). Consistent with this observation, our data found that in the presence of overexpressed β-arrestin1, the cytosolic phosphorylation degree of ERK1/2 was totally enhanced. However, difference in nuclear translocation of phosphorylated ERK1/2 by β-arrestin1 remains to be illustrated. The involvement of ERK1/2 phosphorylation in nNOS activation in a fructose-induced model of hypertension (Cheng et al., 2016) presents feasibility for investigating ERK1/2–nNOS pathway in the current study. Consistent with this hypothesis, our results have proved that the β-arrestin1-induced ERK1/2 phosphorylation was responsible for nNOS activation and NO release in the RVLM. Interestingly, ERK1/2 silencing alone presented no effect on NO production or nNOS phosphorylation in the RVLM of SHRs. Previous evidence has shown that ERK1/2 activation triggered ribosomal S6 kinase (RSK) phosphorylation, which directly phosphorylates nNOS (Song et al., 2007), indicating an indirect regulation of ERK1/2 on nNOS. These findings together predict a direct or indirect link between ERK1/2 and nNOS and suggest a pivotal role of β-arrestin1 in mediating this molecular communication in the RVLM. However, it is a pity that our results showed no changes in RSK expression by β-arrestin1 overexpression in the RVLM, suggesting that RSK signaling pathway may not be involved in β-arrestin1-induced activation of nNOS.

In summary, the present study extends our previous findings to investigate the role of β-arrestin1 on NO release in the RVLM under hypertensive condition. We conclude that (1) an enhancement in NO release is an important cause of β-arrestin1-induced protective response in the RVLM of hypertension, and (2) this effect is underpinned, at least partly, by ERK1/2-mediated nNOS activation. The present study highlights central β-arrestin1 as an important manager for cardiovascular regulation, impelling us to further explore other potential roles of neuromodulators for cardiovascular protection in hypertension.

## Data Availability Statement

The raw data supporting the conclusions of this article will be made available by the authors, without undue reservation.

## Ethics Statement

The animal study was reviewed and approved by the Institutional Animal Care and Use Committee of Naval Medical University.

## Author Contributions

J-CS, XT, and L-JG have contributed equally to conduct the experiments, collect and analyze the data of the study. J-CS drafted the initial version of the manuscript. M-JX and W-ZW provided revisions on the manuscript. All authors have read and approved the final version of the manuscript for publication, agreed to be accountable for all aspects of the work, and ensuring that questions related to the accuracy or integrity of any part are appropriately investigated and resolved. All persons designated as authors qualify for authorship, and all those who qualify for authorship are listed.

## Conflict of Interest

The authors declare that the research was conducted in the absence of any commercial or financial relationships that could be construed as a potential conflict of interest.

## Abbreviations

AAV: adeno-associated virus
BP: blood pressure
ERK1/2: extracellular regulated kinase
GFP: green fluorescent protein
HR: heart rate
L-NAME: N-nitro -L-arginine methyl ester
nNOS: neuronal NO synthase
NO: nitric oxide
RSNA: renal sympathetic nerve activity
RVLM: rostral ventrolateral medulla
SHR: spontaneously hypertensive rat
siRNA: small interfering RNA
WKY: Wistar-Kyoto.
